# Adoption of Evidence-Based Fall Prevention Practices in Primary Care for Older Adults with a History of Falls

**DOI:** 10.3389/fpubh.2016.00190

**Published:** 2016-09-08

**Authors:** Elizabeth A. Phelan, Sally Aerts, David Dowler, Elizabeth Eckstrom, Colleen M. Casey

**Affiliations:** ^1^Department of Medicine, Division of Gerontology and Geriatric Medicine, School of Medicine, University of Washington, Seattle, WA, USA; ^2^Department of Health Services, School of Public Health, University of Washington, Seattle, WA, USA; ^3^Violence and Injury Prevention Program, Utah Department of Health, Salt Lake City, UT, USA; ^4^Program Design and Evaluation Services, Multnomah County Health Department, Oregon Health Authority, Portland, OR, USA; ^5^Division of General Internal Medicine and Geriatrics, School of Medicine, Oregon Health and Science University, Portland, OR, USA; ^6^Providence Health & Services, Portland, OR, USA

**Keywords:** accidental falls/*prevention and control, aged 80, risk assessment/standards, risk factors, medical audit, practice patterns, physicians/*standards

## Abstract

A multifactorial approach to assess and manage modifiable risk factors is recommended for older adults with a history of falls. Limited research suggests that this approach does not routinely occur in clinical practice, but most related studies are based on provider self-report, with the last chart audit of United States practice published over a decade ago. We conducted a retrospective chart review to assess the extent to which patients aged 65+ years with a history of repeated falls or fall-related health-care use received multifactorial risk assessment and interventions. The setting was an academic primary care clinic in the Pacific Northwest. Among the 116 patients meeting our inclusion criteria, 48% had some type of documented assessment. Their mean age was 79 ± 8 years; 68% were female, and 10% were non-white. They averaged six primary care visits over a 12-month period subsequent to their index fall. Frequency of assessment of fall-risk factors varied from 24% (for home safety) to 78% (for vitamin D). An evidence-based intervention was recommended for identified risk factors 73% of the time, on average. Two risk factors were addressed infrequently: medications (21%) and home safety (24%). Use of a structured visit note template independently predicted assessment of fall-risk factors (*p* = 0.003). Geriatrics specialists were more likely to use a structured note template (*p* = 0.04) and perform more fall-risk factor assessments (4.6 vs. 3.6, *p* = 0.007) than general internists. These results suggest opportunities for improving multifactorial fall-risk assessment and management of older adults at high fall risk in primary care. A structured visit note template facilitates assessment. Given that high-risk medications have been found to be independent risk factors for falls, increasing attention to medications should become a key focus of both public health educational efforts and fall prevention in primary care practice.

## Introduction

Falls are the leading cause of unintentional injury-related deaths and non-fatal injuries in people aged 65 years and older (1). Falls predispose to injury, loss of independence, decreased mobility, hospitalization, nursing home placement, and early death (1–3). Each year, accidental falls result in over two million emergency department (ED) visits (1), and fall-related injury care costs exceed $30 billion annually (4). Of particular concern, rates of fall-related ED visits and hospitalizations are increasing (5, 6), and the proportion of older adults in the population is growing, creating an epidemic of falls. Clearly, prevention of falls and the injuries that they cause is a pressing public health issue.

Most falls in community-dwelling older adults result from a combination of risk factors (7–10). A multifactorial approach to assess and manage modifiable risk factors has been identified as an effective intervention for individuals with a history of falls (7–10). However, the extent to which this evidence has penetrated into routine health-care practice in the United States remains unclear (11–18). Data from over a decade ago from community-based primary care practices suggest that translation of fall-prevention evidence into practice was limited, with fall-focused physical examinations and treatment plans present in less than a third of medical records of patients who had sustained a fall (16, 17). More recent evidence suggests that the quality of falls evaluation and management in primary care remains suboptimal (11, 12, 19). The present study, thus, sought to assess the current state of primary care for falls in the United States and identify factors associated with fall risk assessment by primary care providers among persons at high risk of falls.

## Materials and Methods

### Setting

This study was conducted in an outpatient primary care clinic of a large academic medical center in the Pacific Northwest United States. The health system uses an electronic health record (EHR). Primary care providers of the clinic include both medical center faculty and medical residents. The majority of faculty are general internists. Medical residents have their own patient panels. A systematic, clinic-based screening protocol was not in place at the time this study was conducted.

The clinic has a structured note template available for provider use for three types of office visits: Medicare annual wellness visits (a covered Medicare benefit focusing on health promotion), geriatrics consults, and geriatrics “establish care” visits, a patient’s initial appointment with a primary care provider who is a geriatrics specialist. The templates guide systematic evaluation of geriatric conditions; however, their use is left to the discretion of the provider. Structured note templates have been associated with better quality of care for preventive health issues (17, 20, 21).

### Study Sample

Study subjects were outpatients of the clinic, aged 65–95 years, with a documented fall requiring medical treatment or two or more falls within a 12-month period. We focused on those who had already fallen because we hypothesized that this would be the group that would be most likely to receive multifactorial fall risk assessment and management, consistent with national clinical practice guidelines (10). The index fall, defined as the fall after which care practices were examined, was either a fall that resulted in medical care or a fall that was reported during a clinic visit. If more than one fall occurred, the most recent fall within the study period was used as the index fall. The following International Classification of Diseases, 9^th^ Revision (ICD-9) codes were used to determine probable history of a fall (22): 920-924 (contusions with intact skin surfaces), 831-834 (dislocations), E880-E888 (unintentional/accidental fall injuries), V15.88 (history of falls, falls frequently), 802-829 (fractures), and 844-848 (sprains and strains). The study period for purpose of medical records abstraction of included subjects seen in clinic from October 1, 2010 to March 31, 2012. This timeframe was chosen because it coincided with the release of a major national falls guideline but was prior to national initiatives [e.g., Centers for Medicare and Medicaid Services Physician Quality Reporting System (PQRS)] that promote falls screening and management in primary care. We excluded patients who were treated for a fall in an ED or hospital and had no follow-up clinic visit within 3 months after their acute-care episode, patients identified as non-ambulatory during medical record review, and patients with dementia (Alzheimer’s disease, dementia unclassified, vascular dementia, or dementia with Lewy bodies in the EHR problem list), since evidence for benefit of the multifactorial approach with community-dwelling elders with dementia is lacking (10).

There were 42 primary care providers in our study. Of these, 35 were general internists, and 7 were geriatrics specialists (geriatricians, geriatrics-trained advanced practice providers, or internal medicine residents with a primary care panel of adults aged 65+ years). The University of Washington and Oregon Health and Science University institutional review boards approved the study and granted a waiver of consent.

### Data Collection

#### Abstraction Methods

A single researcher (SA) abstracted all primary care office visits for the index fall and the subsequent 3 months. The 3-month time frame was chosen for consistency with quality indicators for assessment of fall-related quality of care that have become standard in the field of older adult health care (23). In addition, the reviewer abstracted all primary care office visits for the subsequent 4–12 months after the index fall coded for: history or risk of fall (V15.88), dizziness and giddiness (780.4), balance problem (781.99), ataxia (334.3), and visits using a structured visit note template (Medicare wellness visits, geriatrics consults, and geriatrics establish care visits). This time frame aligns with guideline recommendations for yearly evaluation of fall risk (10). A review of 10% of charts by a second researcher established inter-rater reliability of 94%. An expert panel of geriatrics specialists, blinded to subject identities, guided the research team in study design, execution, and analysis.

#### Fall Risk Assessments and Interventions

The selection of fall risk factor assessments and interventions abstracted from the EHR was based on current guideline recommendations for the prevention of falls in older adults (9, 10). Table 1 provides assessment definitions and criteria for positive assessments and interventions used in this study. Nine fall-risk factors were abstracted. Seven risk factors included assessments with a corresponding intervention: postural hypotension, lower extremity muscle strength, gait and/or balance, visual acuity, feet and/or footwear, environmental hazards, and vitamin D lab test and/or supplementation. Fall description, the eighth fall risk factor, did not have a corresponding intervention.

**Table 1:** **Definitions of abstracted fall risk assessments and interventions**.

Fall risk assessments	Assessment definition	Criteria for a positive assessment	Intervention defined for a positive risk factor
Detailed description of fall^a^	Documented descriptors of fall: time, circumstance, direction, injuries, symptoms, and other consequences	At least 3 of the 6 descriptors were documented	___^b^
Postural hypotension^a^	Measure BP after lying for 3 min. Repeat BP measurements after 1–3 min standing	A drop in systolic BP of ≥20 mm of mercury or diastolic BP of ≥10 mm of mercury between position changes	Medication adjustment
			Address hydration/diet
			Plan for continued monitoring
Lower extremity muscle strength^a^	Lower extremity manual muscle test	4+/5 or less on manual muscle test	Referral to community exercise class
	Sit to stand ability noted	Difficulty performing sit to stand test due to lower extremity muscle weakness	Recommended participation in a regular exercise program
	Timed Up and Go test	Timed Up and Go ≥15 s	Referral to physical therapy for gait or lower extremity problem
Gait and/or balance^a^	Standardized test, i.e., Timed Up and Go or Romberg test	Timed Up and Go ≥15 s	Referral to physical therapy for gait or lower extremity problem
	Observation of gait or balance	Loss of balance during Romberg test	Referral to community exercise class
	Patient’s report of gait/balance problems	Impaired gait or balance noted by provider	
		Impaired gait or balance reported by patient	
Visual acuity^a^	Vision exam	Documentation of vision deficit/recent change in visual acuity	Ophthalmology or optometry consult
	Reported changes in vision	Ophthalmology or optometry consult	
	Ophthalmology or optometry consult		
Feet and/or footwear^a^	Feet/footwear exam	Foot deformity present	Podiatry consult or monofilament test
	Sensory examination of feet	Inadequate footwear	Address proper foot wear and care of feet
	Podiatry consult or monofilament test	Decreased sensation	
		Podiatry consult or monofilament test	
Environmental Hazards^a^	Discussion of home environment	Home safety hazards identified	Referral for home safety evaluation
			Recommend removal of fall hazards
Vitamin D^a^	Query current vitamin D use	Inadequate vitamin D intake/exposure	Recommend vitamin D supplement of at least 800 IU/day
	Test vitamin D blood levels	Vitamin D lab results <30 ng per ml	25-hydroxy vitamin D levels 30–70 ng/ml
Prescribed medication(s) associated with high risk for fall	___^c^	Prescribed ≥1 medication in Table 2	Medication reduction or change attempted
			Documentation of necessity of the prescription

*BP, blood pressure; IU, international unit*.

*^a^Included in fall risk assessment score*.

*^b^No intervention for fall description*.

*^c^Medication list for prescription of high fall risk meds*.

The ninth risk factor, review of prescription medication, was evaluated only as an intervention, because “medication review” occurred on all patients as an institutional standard of care for all outpatient visits. Chart review of this risk factor was, thus, directed toward identifying patients with whom some action could reasonably have been taken to address high-risk medications. High-risk medications, for purposes of this study, included the following medication classes associated with falls: Benzodiazepines, non-benzodiazepine hypnotics, tricyclic antidepressants, and anticholinergics (see Table 2 for names of medications) (24–26). Charts were reviewed for an appropriate intervention (evidence of a dosage reduction, recommendation to adjust dosage, or documentation of necessity of the prescription) for any of the high-risk medications.

**Table 2:** **High-risk medications included in medical record review (24, 25)**.

**Benzodiazepines**	**Tricyclics**
Chlordiazepoxide	Doxepin
Clonazepam	Amitriptyline
Clorazepate	Nortriptyline
Diazepam	Desipramine
Flurazepam	Imipramine
Estazolam	**Anticholinergics**
Lorazepam	Diphenhydramine
Triazolam	Hydroxyzine
Alprazolam	Meclizine
Midazolam	Cyclobenzaprine
Oxazepam	Methocarbamol
Temazepam	
**Non-benzodiazepine Hypnotics**	
Zaleplon	
Zolpidem	
Eszopiclone	

Two variables (vision; feet/footwear) were counted as both fall risk factor assessments and interventions, because assessment was inferred as having occurred on the basis of a specific action (e.g., referral) being taken. Aspects of routine medical evaluation that were not specific to falls (e.g., general physical examination, neurological examination, heart rate and rhythm) were not included in the abstraction.

#### Fall Risk Assessment Score

For analysis purposes, a fall-risk assessment score was created for each patient by summing eight fall risk assessments (excluding medication review) performed by PCPs over the 12 months after the index fall. Scores ranged from 0 to 8, with higher scores representing more risk factors assessed and, therefore, higher guideline adherence.

#### Independent Variables

##### Falls and Fall-Related Health-care Use

The number of falls and ED visits and hospitalizations for falls or fall-related injuries were abstracted for 12 months subsequent to the index fall. The number of falls included the index fall, patient-reported falls, and medically attended falls recorded in the EHR. All ED care and hospitalizations within the medical center were recorded; care for a fall at another institution was included if noted in the EHR.

##### Primary Care Visits

The number of clinic visits within 12 months following the index fall was counted. We also counted a subset of clinic visits that specifically addressed falls, fall risk, or medical consequences of the index fall. These visits were either coded for history or risk of fall, dizziness and giddiness, balance problem, unsteady or abnormal gait, and ataxia or coded for musculoskeletal injuries that matched codes associated with the index fall, for example, hip fracture (821.00) or shoulder pain (719.41).

##### Comorbidities

Comorbidities were those identified in prior research as risk factors for falls or fall-related injury: cardiovascular disease, history of cerebrovascular accident, mild cognitive impairment, depression, diabetes mellitus, gait disturbance, hypertension, incontinence, osteoarthritis, osteoporosis, Parkinson’s disease, vertigo, and visual impairment (8, 27, 28). Comorbidities added to the EHR patient problem list by health-care providers prior to, or within 3 months of the index fall, were abstracted.

### Data Analysis

Patients of general internists and geriatrics specialists were compared on baseline demographic, health, and fall-related health-care utilization. Chi-square and independent-samples *t*-tests were used to test for between-group differences on these variables. Bivariate two-tailed Pearson correlation coefficients were calculated for the primary dependent variable, i.e., the fall risk assessment score, and independent variables hypothesized to influence the number of assessments performed. Results were considered statistically significant at *p* < 0.05. To test for independent effects, variables showing significant associations at the *p* = 0.05 level in the bivariate analysis were entered simultaneously in a multiple regression model predicting the fall risk assessment score. The data were analyzed using SPSS software, version 22.0 (SPSS Inc., Chicago, IL, USA).

## Results

### Patient Characteristics and Fall-Related Health-care Utilization

A total of 256 patients were identified as having fallen during the study period. Of these, 140 were ineligible, for the following reasons: 99 patients had no clinic visit within 3 months after their fall, 4 were non-ambulatory, and 37 had documented dementia. The remaining 116 patients met eligibility criteria and were included in the analysis.

Table 3 shows baseline demographic and health characteristics, and fall-related health-care utilization of the 116 patients, overall and by PCP specialty. Their mean age was 79 ± 8 years, 68% were female, and 10% were non-white. During the 12-month abstraction period, beginning with the index fall, 249 falls were recorded; 186 (75%) were reported during a primary care office visit, 45 (18%) resulted in ED care, and 18 (7%) required hospitalization. Eighty percent of patients had 1 or 2 falls, 16% had 3 to 6 falls, and 4% had more than 10 falls.

**Table 3:** **Patient baseline demographic and health characteristics, and fall-related health-care utilization, overall and by provider specialty**.

Characteristic	Total sample (*N* = 116)	General internist subgroup (*n* = 86)	Geriatrics specialist subgroup (*n* = 30)	*p* value
Age, years, mean ± SD	78.6 ± 7.7	77.2 ± 6.9	82.7 ± 8.6	0.001
Female, %	68.0	65.0	77.0	0.35
Non-white, %	9.5	12.8	0	0.09
Medications, number, mean ± SD	13.0 ± 6.1	12.6 ± 6.2	14.3 ± 5.8	0.20
Comorbidities, number, mean ± SD	2.1 ± 1.5	2.0 ± 1.4	2.6 ± 1.6	0.05
**Comorbidities, %^a^**				
Cerebrovascular accident^b^	8.6	8.1	10.0	1.00
Mild cognitive impairment^c^	12.9	9.3	23.3	0.10
Depression^d^	39.7	36.0	50.0	0.26
Diabetes mellitus	20.7	24.4	10.0	0.16
History of fall(s) or gait disturbance^e^	38.8	36.0	46.7	0.42
Osteoporosis	27.6	23.3	40.0	0.13
Parkinson’s disease	9.5	7.0	16.7	0.23
Vertigo^f^	7.8	5.8	13.3	0.35
Visual impairment^g^	47.4	46.5	50.0	0.91
Average number of falls ± SD^h^	2.2 ± 2.3	2.2 ± 2.6	2.1 ± 1.2	0.90
Primary care office visits, mean ± SD^h^	6.4 ± 3.9	6.2 ± 4.2	6.9 ± 3.1	0.42
Primary care office visits addressing falls, fall risk, or medical complications of fall, mean ± SD^h^,^i^	1.8 ± 1.2	1.9 ± 1.3	1.6 ± 0.8	0.16
Primary care office visit used structured note template	13.8 ± 3.5	9.3 ± 0.3	26.7 ± 0.5	0.04
Fall-related emergency department visit, %^h^	34.5	30.2	46.7	0.38
Fall-related hospitalizations, %^h^	15.5	17.4	10.0	0.29

*^a^Comorbidity added to EHR patient problem list prior to, or within 3 months, of the index fall*.

*^b^Transient ischemic attack, cerebral infarct, cerebrovascular disease*.

*^c^Memory loss*.

*^d^Bipolar disorder, dysthymia*.

*^e^Abnormal gait, ataxia, balance problem, falls frequently, at risk for falls*.

*^f^Dizziness, giddiness, long-standing (≥6 months) benign paroxysmal positional vertigo*.

*^g^Cataract, poor vision post-cataract removal, diabetic retinopathy, glaucoma, macular degeneration, legal blindness, senile nuclear sclerosis*.

*^h^Within 12 months post index fall, including index fall*.

*^i^Primary care office visits coded for history or risk of fall (V15.88), dizziness and giddiness (780.4), balance problem (781.99), unsteady/abnormal gait (781.2), ataxia (334.3), or medical consequences of a fall matching office visit codes for index fall*.

The mean number of clinic visits over the 12-month abstraction period was 6.4 (range 1–20) (Table 3). Roughly one-third of 739 primary care office visits addressed falls, fall risks, or medical consequences of a fall. Patients seen by geriatrics specialists were significantly older and had a greater number of comorbidities compared to those seen by general internists. Geriatrics specialists were significantly more likely to use a structured note template to document the clinic visit. There were no other significant between-specialty differences for the variables shown in Table 3.

### Fall Risk Factor Assessments and Interventions

Results for the documented fall risk factor assessments and interventions are shown in Table 4. Performance of fall risk factor assessment ranged from 24 (home safety) to 78% (vitamin D). Lower extremity muscle strength, gait/balance, and vision assessments were each performed in about half of the study sample. One-third of the gait/balance assessments were a standardized performance test, a Timed Up and Go or Romberg test. Referral to a vision specialist accounted for over half of the vision assessments (35 of 63) and interventions (35 of 56). Monofilament examinations accounted for over half of the feet/footwear assessments (17 of 33) and interventions (17 of 26).

**Table 4:** **Fall-risk assessments and interventions performed with study sample (*N* = 116)**.

Fall risk assessment	Assessment performed (%)	Risk factor present (%)	Intervention(s) recommended (%)
Fall description in medical record	78 (67.2)	___^a^	___^b^
Postural hypotension	35 (30.2)	8 (22.9)	7 (87.5)
Vision (during primary care office visit, ophthalmology/optometry consult or eye clinic visit)	63 (54.3)	57 (90)	56 (98.2)
Feet/footwear (during primary care office visit, monofilament exam or podiatry consult)	33 (28.4)	29 (87.9)	26 (89.7)
Lower extremity muscle strength and PT referral	59 (50.9)	18 (30.5)	16 (88.9)
**Gait/balance**	62 (53.4)	27 (42.9)	26 (96.3)
Gait/balance problem and PT referral		27 (42.9)	24 (88.9)
Gait/balance problem and exercise recommended		27 (42.9)	15 (55.6)
Gait/balance problem and assistive device recommended		27 (42.9)	10 (37.0)
Home/environmental safety (provider recommendations or home health referral)	Combined assessment and intervention		28 (24.1)
Vitamin D ≥800 IU/day prescribed or 25-hydroxy vitamin D lab test	Combined assessment and intervention		91 (78.4)
High-risk medication	___^c^	29 (25.0)	6 (20.7)

*PT, physical therapist; IU, international unit*.

*^a^At least one fall had occurred in all participants in the study sample*.

*^b^No intervention for fall description*.

*^c^All visits included a medication review as part of routine care; fall-related medication assessment could not differentiated for purposes of the study*.

Interventions were prescribed most frequently (78–98% of the time) for the following risk factors, given here in order of increasing frequency: vitamin D, postural hypotension, lower extremity strength, feet/footwear, gait/balance, and vision. Two risk factors – medications and home safety – were addressed less frequently. Of the 29 patients whose medication list included a high-risk medication at the time of their index fall, 6 (21%) had medications addressed post-fall. A home safety evaluation was ordered for 24% of the study sample.

### Fall Risk Assessment Score and Correlation with Independent Variables

The mean fall risk assessment score for all patients was 3.9 ± 1.7, indicating that, on average, providers performed about half of the recommended assessments over the 12 months following an index fall. Geriatrics specialists performed significantly more assessments than general internists (4.6 vs. 3.6, respectively, *p* = 0.007).

Table 5 shows correlations of the fall risk assessment score with patient characteristics and clinic treatment variables and results of the multiple regression analysis. The number of clinic visits within 12 months of the index fall showed a significant correlation with the score (Pearson *r* = 0.37, *p* < 0.001). Most patients (19 of 21) who received six to eight assessments had five or more office visits. Patients who underwent an evaluation in which the PCP used a structured visit note template (*n* = 16) received more assessments, on average, than those who did not (5.1 vs. 3.7, *p* = 0.002). Number of prescribed medications, depression, diabetes, number of falls within 12 months post index fall, and geriatrics specialty were also significantly correlated with the score. When entered simultaneously in a multiple regression model predicting the fall risk factor assessment score, the number of office visits, use of a structured note template, diagnosed diabetes, number of falls, and geriatrics specialty remained independent predictors (Table 5). The model’s coefficient of determination (*R^2^*) was 0.344.

**Table 5:** **Bivariate correlations and multiple regression of fall risk assessment score^a^ by independent variables**.

Variable	Pearson correlation coefficient	*p* value	Multiple regression *p* value
Gender (female)	0.034	0.715	___^b^
Age at fall	−0.004	0.962	___^b^
Number of prescribed medications	0.273	0.003	0.184
**Comorbidities**^c^			
Cerebrovascular accident^d^	0.058	0.534	___^b^
Mild cognitive impairment^e^	0.058	0.534	___^b^
Depression^f^	0.253	0.006	0.355
Diabetes mellitus	0.259	0.005	0.008
History of fall(s) or gait disturbance^g^	0.172	0.066	___^b^
Osteoporosis	0.046	0.624	___^b^
Parkinson’s disease	0.177	0.058	___^b^
Vertigo^h^	0.077	0.409	___^b^
Visual impairment^i^	0.170	0.068	___^b^
Number of falls^j^	0.225	0.015	0.029
Number of primary care office visits^i^	0.369	<0.001	0.032
Geriatrics specialist^k^	0.248	0.007	0.021
Structured visit note template^l^	0.289	0.002	0.003

*^a^Number of fall risk factor assessments performed by primary care provider, range, 0–8*.

*^b^Variable not included in the multiple regression model*.

*^c^Comorbidity added to EHR patient problem list prior to, or within 3 months, of the index fall*.

*^d^Transient ischemic attack, cerebral infarct, cerebrovascular disease*.

*^e^Memory loss*.

*^f^Bipolar disorder, dysthymia*.

*^g^Abnormal gait, ataxia, balance problem, falls frequently, at risk for falls*.

*^h^Dizziness, giddiness, long-standing (≥6 months) benign paroxysmal positional vertigo*.

*^i^Cataract, poor vision post-cataract removal, diabetic retinopathy, glaucoma, macular degeneration, legal blindness, senile nuclear sclerosis*.

*^j^Within 12 months post index fall, including index fall*.

*^k^Geriatricians, geriatrics-trained advanced practice providers, or internal medicine residents with a patient panel of older adults (aged 65+)*.

*^l^Medicare Wellness visits, geriatrics consults, and geriatrics establish care visits*.

## Discussion

### Summary of Main Results

This study assessed the extent to which primary care practice for fall prevention aligns with current evidence. Translation of fall-prevention evidence into practice in our study sample appears modestly improved since the last decade, with just over half (54%) of individuals at high risk of future falls (based on history of a fall), receiving at least half of the recommended assessments within 12 months. Once identified, risk factors were usually addressed (73% on average). Notable exceptions were home safety and medications, addressed with 24 and 21%, respectively. Use of a structured visit note template, geriatrics specialty, and number of office visits independently predicted PCP performance of fall risk assessments.

### Comparisons with Other Studies

In prior observational research of fall risk evaluation and management in primary care practice, two studies used medical record review (11, 16) and two others used physician self-report (12, 19); performance of most fall risk assessments was 50% or less. Our study found that some fall-risk factors – namely postural hypotension, visual acuity, and gait and balance – were assessed over twice as frequently compared to the earlier United States, chart-based study (16) (postural hypotension, 30 vs. 6%; visual acuity, 54 vs. 25%; gait and balance, 18 vs. 10%). Health-care providers in our study also appeared to prescribe interventions more frequently for identified fall risks (73 vs. 14–55% in the other studies) (11, 16, 17).

Why did the present study demonstrate more frequent assessments and interventions compared to prior studies? Possible explanations for this finding include the publication of a number of systematic reviews and updated guidelines on fall prevention in major medical journals in recent years (7–10). Inclusion of geriatrics specialists in our study sample of primary care providers and the presence of these specialists in the clinic setting would tend to bias toward demonstrating more comprehensive care for geriatrics conditions such as falls. Geriatrics specialists in our study performed more fall risk assessments than general internists. This is not surprising, since geriatrics specialists are trained to address complex, multifactorial health issues, and falls are a recognized geriatric syndrome (29, 30). This finding is also consistent with results from an observational study in which geriatricians scored higher than generalists on assessment of geriatric syndromes (31).

Another possible explanation for the higher frequency of most assessments and interventions could be differences in definition of what “counted” as a fall risk factor assessment or intervention. However, we modeled our assessment definitions after the only other United States-based, observational study examining fall-related quality of care that used medical record review (16). When compared with the definitions used in that study (16), they were quite comparable (e.g., documented drop in blood pressure defined orthostatic hypotension; vision exam or notation in chart regarding vision defined visual acuity; home hazard assessment and modifications; PT referral or exercise or assistive device recommended if gait/balance problem identified).

The finding that an increased number of fall risk factor assessments occurred with a greater number of office visits fits with prior research on geriatric syndrome care: more visits equate with more “opportunities” to deliver preventive care (31).

As described in our “Materials and Methods,” “medication review” occurred on all patients as an institutional standard of care for all outpatient visits at our institution. In the era of EHRs, which typically prompt medication review prior to permitting a provider to sign his/her visit note in a patient’s chart, such medication reviews are increasingly likely to occur for all patients, at every clinic visit. It is important to note that these “standard of care reviews” can be accomplished within an EHR with a click of a button and do not (as our study demonstrated) necessarily prompt more rational medication prescribing.

### Recommendations for Practice: Use Structured Visit Note Templates

Use of a structured visit note template was highly significantly associated with the performance of fall-risk factor assessments, even after controlling for potentially confounding variables (including geriatrics specialty). One example of a visit conducive to a structured visit note template is the Medicare annual wellness visit (32). The health risk assessment, a requirement of the visit, includes three fall risk factor assessments: medication review, fall history/fear of falling, and home safety questions (33). In the context of a busy primary care practice, use of a structured visit note template may facilitate completion of fall risk factor assessments (17, 21, 34).

### Recommendations for Practice: Recommend Home Safety Evaluations

Despite the effectiveness, cost-benefit, and cost-effectiveness of home safety evaluations in reducing falls (6, 35, 36), referral for a home safety evaluation occurred in only 24% of the study sample. This finding closely resembles findings (18%) from a patient cohort in the Netherlands (11) and findings from the control group (16.7%) of a practice-change, primary-care-based intervention trial aimed at improving the quality of care for geriatric syndromes (18). Taken together, these data suggest a specific target for care improvement. Options for the provision of home safety evaluations for non-homebound individuals vary across the United States, but for the most part are not widely available. In some areas, physical or occupational therapists provide Medicare Part B services in the home (37). In other areas, emergency medical service (EMS) providers offer this service. One example of a well-developed EMS program is One Step Ahead (38). However, this program and others like it in the United States have limited reach and uncertain long-term viability, given that they tend to rely on short-term grant funding. Home modifications represent a low-cost, high-return intervention to reduce fall injuries (35, 36). Going forward, making home safety assessment and modifications a covered health insurance benefit for all older adults at high risk of falls offers the opportunity to reduce falls and their associated health-care costs. Meanwhile, PCPs and their staff are encouraged to research available options in their area and order home safety evaluations by their rehabilitation colleagues for their patients at high risk for falls.

### Recommendations for Practice: Increase Attention to High-Risk Medications

Our results suggest a need for increased attention to the contribution of medications to falls. One-quarter of our sample was on a medication associated with falls, and only 21% of these had their prescription dose-reduced or discontinued or documentation of continued need for the medication after their fall. A recent observational study that focused on a single class of fall-risk-increasing medication (benzodiazepines) (11) found an intervention to decrease or stop the medication in 49% of cases; another study that examined psychopharmacy found a rate of 28% (12), which is very comparable to ours. Taken together, these findings suggest that medications represent another key focus for care improvement in primary care.

Several evidence-based resources are available to guide prescribing practices with older adults: The AGS’ Beers criteria for potentially inappropriate medication use for older adults (39); the Screening Tool of Older Persons’ potentially inappropriate Prescriptions (STOPP) criteria (40, 41); and the Screening Tool to Alert doctors to Right Treatments (START) criteria (40, 41). As part of the Centers for Disease Control and Prevention (CDC) Stop Elderly Accidents Deaths and Injuries (STEADI) initiative, a brief training module for health-care providers on the role of medication review and reduction as a key, evidence-based strategy for reducing falls among older adults will soon be available for continuing education credit (42).

### Recommendations for Practice: Use the STEADI Materials

In order to assist PCPs in adopting new practice patterns, the CDC developed STEADI (42). STEADI is a comprehensive set of materials that provides a foundation to systematically evaluate and address fall risk. STEADI includes an algorithm to assess fall risk, tips for integrating fall risk management into clinical practice, assessment tools for modifiable fall-risk factors, descriptions of interventions, and patient education materials. It is a systematic, evidence-based, accessible, and free resource for PCPs and their practice teams to evaluate and manage their patients’ fall risk.

### Recommendations for Practice: Increase Public Health Messaging about Falls and their Preventability

Little work has, thus far, been conducted at the national level to raise public awareness of the fact that falls are often preventable. One state-level project to disseminate fall-prevention evidence involved a multicomponent dissemination strategy that included fall-prevention messaging distributed *via* a number of communication channels (e.g., public service announcements on radio and television) to raise awareness (43). Similar to public health messages regarding other acute, potentially life-threatening events (e.g., myocardial infarction, stroke), messages that convey that falls occur frequently but are often preventable may help de-stigmatize their occurrence and encourage people who are falling to take steps to address their modifiable fall-risk factors.

### Limitations and Strengths

This study has several limitations. First, data were collected using medical records, with the limitations inherent in this type of record-based review (i.e., lack of documentation of actions taken). However, record-based review is superior to self-report of practice, upon which other published studies on this topic have relied (12, 15). Second, data on fall-risk factor assessment and management was abstracted after, but not prior to, the index fall. Therefore, management of fall risks that occurred in close proximity, but prior to, the index fall was not captured. Third, the 116 patients were a convenience sample generated using administrative data to select cases that were probable falls. This selection process may have missed falls that did not receive medical attention. However, this approach would tend to bias findings toward an over-estimation of health-care quality, meaning that the data we present herein likely represents “best-case scenario” for care quality for falls. Our requirement that a patient have a clinic visit within 3 months of a fall-related health-care episode was used because benchmark quality indicators document that a 3-month time frame for assessment following a fall is appropriate (23); however, this criterion resulted in exclusion of nearly half of our initial sample of patients with a fall and may have led to selection bias (e.g., either a more or less frail sample). Fourth, the number of providers whose practices were examined was fairly small; studies involving larger practices would be worthwhile. Fifth, findings may not generalize to non-academic (community-based) practices. The clinic in which our study was conducted most likely resembles other primary care internal medicine clinics at academic health centers, except for the geriatrics-trained health professionals who were part of the practice mix. Sixth, given that the study site had a well-developed EHR, findings may not be reflective of health care received by community-dwelling older adults in practice settings that either do not use EHRs or whose EHRs are not integrated with a multi-disciplinary health-care organization. However, results should be generalizable to other academic health center practices with established EHRs. Academic health centers are responsible for training heath care providers of the future and so should be in the forefront of modeling and teaching evidence-based practices. A notable strength is that our findings are unlikely to have been affected by any unmeasured contextual factors, such as clinic staff involvement in falls screening or institutional metrics promoting benchmarks related to falls screening, as there was no formal fall risk screening and management protocol in place at the time the study was conducted.

In summary, our study suggests that there may be ongoing opportunities to improve primary care of older adults with a history of falls. This can be accomplished through assessment and management of modifiable fall-risk factors, including home safety and medications. Ours is the first study of United States-based fall management practices in over a decade, subsequent to the advent of EHRs and to the publication of several notable evidence-based guidelines. Structured visit note templates and newly available public health resources can help practices restructure and optimize their approach to delivering preventive care for patients at risk for falls, a largely preventable, high-cost condition.

## Author Contributions

Conception and design of the study: EP, SA, EE, and CC. Acquisition of data, analysis, and interpretation of data: EP, SA, DD, EE, and CC. Drafting the article or revising it for important intellectual content: EP, SA, DD, EE, and CC. Final approval of the version to be published: EP, SA, DD, EE, and CC. Accountability for accuracy and integrity of the work: EP, SA, DD, EE, and CC.

## Conflict of Interest Statement

The authors declare that the research was conducted in the absence of any commercial or financial relationships that could be construed as a potential conflict of interest.
