# Corrigendum: Adoption of Evidence-Based Fall Prevention Practices in Primary Care for Older Adults with a History of Falls

**DOI:** 10.3389/fpubh.2016.00255

**Published:** 2016-11-16

**Authors:** Elizabeth A. Phelan, Sally Aerts, David Dowler, Elizabeth Eckstrom, Colleen M. Casey

**Affiliations:** ^1^Department of Medicine, Division of Gerontology and Geriatric Medicine, School of Medicine, University of Washington, Seattle, WA, USA; ^2^Department of Health Services, School of Public Health, University of Washington, Seattle, WA, USA; ^3^Violence and Injury Prevention Program, Utah Department of Health, Salt Lake City, UT, USA; ^4^Program Design and Evaluation Services, Multnomah County Health Department, Oregon Health Authority, Portland, OR, USA; ^5^Division of General Internal Medicine and Geriatrics, School of Medicine, Oregon Health and Science University, Portland, OR, USA; ^6^Providence Health & Services, Portland, OR, USA

**Keywords:** accidental falls, aged, aged 80 and over, risk assessment standards, medical audit

## Conflict of Interest Statement

The author declares that the research was conducted in the absence of any commercial or financial relationships that could be construed as a potential conflict of interest.

